# Therapeutic plasma exchange in hypertriglyceridemic patients

**DOI:** 10.3906/sag-1811-105

**Published:** 2019-06-18

**Authors:** Ilgın YILDIRIM ŞİMŞİR, Utku Erdem SOYALTIN, Banu SARER YÜREKLİ, Mehmet ERDOĞAN, Şevki ÇETİNKALP, Füsun SAYGILI, Ayhan DÖNMEZ, Ahmet Gökhan ÖZGEN

**Affiliations:** 1 Department of Internal Medicine, Division of Endocrinology and Metabolism Disorders, Faculty of Medicine, Ege University, İzmir Turkey; 2 Department of Internal Medicine, Division of Hematology, Faculty of Medicine, Ege University, İzmir Turkey

**Keywords:** Hypertriglyceridemia, pancreatitis, apheresis, therapeutic plasma exchange, fresh frozen plasma

## Abstract

**Background/aim:**

High triglyceride (TG) levels are associated with increases in atherosclerotic cardiovascular disease (CVD), hepatic steatosis, and pancreatitis. Acute pancreatitis is a condition with high mortality. Therapeutic plasma exchange (TPE) in the treatment of hypertriglyceridemic pancreatitis (HTGP) is a rapid and effective treatment modality. In this study, the results of TPE were evaluated and the frequency of lipoprotein lipase (LPL) mutation in these patients was determined.

**Materials and methods:**

TPE was performed in 31 patients with HTGP at the Adult Therapeutic Apheresis Center.

**Results:**

A TG level under 500 mg/dL was achieved by applying apheresis at a median of 2 times (IQR 2–2, min 1, max 6) in the 31 cases. LPL mutation was detected in 8 (25.8%) of the 31 hypertriglyceridemia cases. When TG levels before and after TPE were evaluated, the mean TG level before TPE was significantly higher (3132 ± 1472 mg/dL) than the mean TG level afterwards (948 ± 465 mg/dL, P < 0.001). This result represented a decrease of 69.7% TG after TPE.

**Conclusion:**

TPE is a safe, fast, and effective treatment modality in experienced centers.

## 1. Introduction

Hypertriglyceridemia (HTG) is a disorder of lipid metabolism that is caused by increased dietary intake, excessive synthesis in the liver, impaired metabolism, or a combination of these effects [1]. Triglycerides (TGs) are one of the human body’s main energy sources. However, high TG levels are associated with increases in atherosclerotic cardiovascular disease (CVD), hepatic steatosis, and pancreatitis [2]. 

Following alcohol consumption and cholelithiasis, HTG is the third most common cause of acute pancreatitis. Between 1% and 10% of all acute pancreatitis cases and 56% of pancreatitis cases during pregnancy are secondary to HTG [2,3]. 

In HTG, TGs are destroyed by pancreatic lipases, which cause the release of free fatty acids and subsequent lipotoxicity, leading to chemical pancreatitis [4]. There is a known positive correlation between HTG levels and the risk of pancreatitis. When TG levels are >1000 mg/dL, the risk of acute pancreatitis is 5%; however, the risk is 10%–20% when TG levels are >2000 mg/dL [5,6]. Despite this fact, there are no standard predefined TG values for pancreatitis development. 

Insulin, heparin, and apheresis are among the 3 treatment methods approved for the treatment of hypertriglyceridemic pancreatitis (HTGP). The American Society for Apheresis has reported that aphaeresis in acute pancreatitis secondary to HTG should be classified as indication category III [7]. Those treatments classified as category III have thus far had inadequate assessments, and therefore, it has been recommended that individualized apheresis should only be applied in addition to other therapeutic treatments [7]. According to the 2016 Guideline, therapeutic plasma exchange (TPE) treatment should be performed every 1–3 days by targeting TG levels <1000 mg/dL (ideally <500 mg/dL) in acute pancreatitis secondary to HTG [7]. 

Like any invasive procedure, TPE has complications [7–9]. Bleeding, hematoma, or infection may occur with catheter use. In addition, patients with pneumothorax may also experience thrombosis, hypocalcemia, hypotension, and transfusion reactions due to the procedure. Furthermore, angiotensin-converting enzyme inhibitors may cause anaphylaxis, while respiratory distress and immunosuppression may occur due to transfusion-mediated diseases, air embolisms, pulmonary edema, and/or pulmonary embolisms [8,9]. 

Lipoprotein lipase (LPL) gene mutation is one the most common causes of primary HTG. It was aimed herein to determine the frequency of LPL mutation in a population with severe HTG.

Acute pancreatitis has a high mortality rate. Quick treatment approaches can be life-saving. In the current study, the results of TPE, which is a rapid, effective, and safe treatment modality in patients with pancreatitis secondary to HTG, were evaluated, and the frequency of LPL mutation in these patients was determined.

## 2. Materials and methods 

In this study, the records of 31 hypertriglyceridemic (HTG) patients who underwent TPE between 2012 and 2018 at the Adult Therapeutic Apheresis Center of Ege University, Faculty of Medicine, were evaluated retrospectively. 

In accordance with the protocol of the Adult Therapeutic Apheresis Center, patients with HTGP, patients who were not able to use antihyperlipidemic drugs due to their side effects, or inefficient and pregnant patients with HTG were chosen for TPE. Inclusion criteria were based on the Ranson criteria and Balthazar score.

Inclusion criteria for TPE were:

· HTG patients with lactic acidosis

· HTG patients with a white blood cell count >16.000/mm3

· HTG patients with a blood glucose level >200 mg/dL

· HTG patients with lactate dehydrogenase >350 IU/L

· HTG patients with aspartate aminotransferase >250 U/L

· HTG patients with multiorgan failure (e.g., renal failure)

· HTG patients with pancreatic necrosis or fluid collections on computerized tomography

Exclusion criteria for TPE were:

· HTG patients who could not tolerate or approve central line placement

· HTG patients who were actively septic or hemodynamically unstable 

· HTG patients with hypocalcemia 

· HTG patients who were currently using angiotensin-converting enzyme inhibitors

For each patient, the amount of plasma to be removed via TPE was determined by the physician, according to the clinical situation. The patient’s plasma volume was calculated based on nomograms generated via information about the patient’s sex, height, weight, and hematocrit level. Fresh frozen plasma was used as a replacement fluid, each time, in all of the cases, at a volume of 1–1.5 times the calculated total plasma volume. The blood flow rate was set between 55 and 70 mL/min. TPE was performed via central venous access using a Baxter CS 3000 blood cell separator (Baxter Healthcare, Deerfield, IL, USA). Vital signs were monitored at the beginning and end of each procedure, and patients were monitored for adverse events during the apheresis procedures. Before each apheresis procedure, written informed consent was obtained from each patient after the procedural risks were explained in detail. Each apheresis session lasted for 2.5–3 h. TPE was administered daily until almost under 500 mg/dL TG was achieved. 

The patient files were evaluated retrospectively. Results with positive LPL gene mutation were noted. All exon and exon-intron compounds that encoded the LPL gene were evaluated by sequencing at the Medical Genetic Laboratory. The results were screened retrospectively.

For the statistical analysis, the data were recorded using the SPSS version 20 (IBM Corp., Armonk, NY, USA). The descriptive statistics in this study were presented as numbers (N) and percentages (%). Prior to the statistical analyses, the data were analyzed to determine whether they were normally distributed. If so, parametric tests were used, and if not, non-parametric tests were used. The Wilcoxon test was used to determine whether there was a significant difference between the first and last medians in cases where the data from 2 independent groups were not normally distributed. In cases where the data were normally distributed, a t-test was used to determine whether the differences in the averages of these groups were significant. Statistical significance was accepted as P < 0.05.****

## 3. Results 

Of the 31 patients in the study, 20 (64.5%) were female and 11 (35.5%) were male. The median and interquartile range (IQR) of their ages was 40 years (30–49). LPL mutation was detected in 8 (25.8%) of the patients. A TG level under 500 mg/dL was achieved by applying apheresis at a median of 2 times (IQR 2–2, min 1, max 6). Three patients (9.7%) experienced complications related to apheresis [catheter infection in 2 patients (66.7%) and catheter thrombosis in 1 patient (33.3%)]. The mean body mass index (BMI) of the patients was 27.0 ± 5.7 kg/m2. All of the patient data are shown in Table 1. The mean Ranson score upon hospital admission was 3.

**Table 1:** Patient data (n: 31).

Variables	Median [IQR] or mean ± SD data of the diabetic patients before TPE (n: 20)	Median [IQR] or mean ± SD data of the non-diabetic patients before TPE (n: 11)	Statistical test and P-values
Total cholesterol (mg/dL)	475 ± 214	429 ± 191	t = –0.578P = 0.555
TGs (mg/dL)	3108 ± 1509	3176 ± 1473	t = 0.121P = 0.905
HDL cholesterol (mg/dL)	30 [18–50]	35 [21–44]	U = 105.500P = 0.852
Non-HDL cholesterol (mg/dL)	442 ± 198	392 ± 168	t = –0.704P = 0.487
LDL cholesterol (mg/dL)	96 ± 51	68 ± 48	t = –1.510P = 0.142
Apo A1 (mg/dL)	99 [79–128]	121 [104–163]	U = 56.500P = 0.082
Apo B (mg/dL)	101 [91–112]	92 [75–139]	U = 90.000P = 0.869
Lipoprotein (a) (nmol/L)	13 [9–29]	9 [7–46]	U = 78.500P = 0.468
25-OH vitamin D (ng/mL)	14 [11–22]	17 [8–20]	U = 105.500P = 0.852
BMI (kg/m2)	28.9 ± 5.7	23.4 ± 3.9	t = –2.878P = 0.007

The mean biochemical results of the 31 patients were as follows: total cholesterol 459 ± 204 mg/dL, TG 3132 ± 1472 mg/dL, HDL cholesterol 34 ± 21 mg/dL, non-HDL cholesterol 424 ± 187 mg/dL, LDL cholesterol 86 ± 51 mg/dL, apolipoprotein (apo) A1 116 ± 43 mg/dL, apo B 105 ± 35 mg/dL, lipoprotein (a) 24 ± 27 nmol/L, 25-OH vitamin D 16 ± 7 ng/mL. These biochemical data are presented in Table 2.

**Table 2:** Mean ± SD biochemical data of all of the groups.

Variables	Findings
Female (n, %)	20 (64.5)
Pregnant (n, %)	5 (25)
Age (years) (median [IQR])	40 (30–49)
Diabetic (n, %)	20 (64.5)
Genetic mutations (n, %)Positive LPL mutationNegative LPL mutation	8 (25.8)23 (74.2)
Number of plasmapheresis sessions (median [IQR])	2 (2–2)
Complication (n, %)	3 (9.7)
Hospitalization time (days)	14 ± 6
BMI (N: 18.5–24.9 kg/m2)	27.0 ± 5.7

When TG levels before and after TPE were evaluated, the mean TG level before TPE was significantly higher (3132 ± 1472 mg/dL) than the mean TG level after TPE (948 ± 465 mg/dL, P < 0.001). This result represented a decrease of 69.7% TG after TPE. TG values before and after TPE are presented in Table 3. 

**Table 3:** TG levels before and after TPE.

Variables	Mean ± SD data of all of the groups (n: 31)
Total cholesterol (N < 200 mg/dL)	459 ± 204
TGs before TPE (N < 150 mg/dL)	3132 ± 1472
HDL cholesterol (N > 60 mg/dL)	34 ± 21
Non-HDL cholesterol (N < 160 mg/dL)	424 ± 187
LDL cholesterol (N < 130 mg/dL)	86 ± 51
Apo A1 (N: 104–202 mg/dL)	116 ± 43
Apo B (N: 66–133 mg/dL)	105 ± 35
Lipoprotein (a) (N < 75 nmol/L)	24 ± 27
25-OH vitamin D (N: 30–80 ng/mL)	16 ± 7

When the 8 patients (25.8%) with positive LPL mutation were compared with the 23 (74.2%) patients without the LPL mutation, there were no significant differences between them with regards to total cholesterol, TGs, HDL cholesterol, non-HDL cholesterol, LDL cholesterol, apo A1, apo B, lipoprotein (a), 25-OH vitamin D, and BMI. Of the patients with LPL mutations, three (37.5%) were diabetic, and their mean hemoglobin A1c (HbA1c) was 8.8%. These data are shown in Table 4. 

**Table 4:** Median and IQR biochemical data of patients with and without LPL mutation.

	Median [IQR] or mean ± SD TG before TPE	Median [IQR] or mean ± SD TG after TPE	Statistical test and P-values
All patients (n: 31)	3132 ± 1472	948 ± 465	t = 8.871P < 0.001
Female patients (n: 20)	2860 ± 1450	1015 ± 474	t = 6.944P < 0.001
Male patients (n: 11)	3626 ± 1446	827 ± 443	t = 6.356P < 0.001
Diabetic patients (n: 20)	2829 [2051–4361]	813 [675–1110]	Z = –2.934P = 0.003
Nondiabetic patients (n: 11)	3176 ± 1473	944 ± 501	t = 6.941P < 0.001
Pregnant patients (n: 5)	2546 [1996–3430]	832 [478–1536]	Z = –2.023P = 0.043
Nonpregnant female patients (n: 15)	3219 ± 1560	944 ± 454	t = 8.080P < 0.001
Patients with LPL mutation (n: 8)	2990 ± 1496	1044 ± 385	t = 8.253P = 0.010
Patients without LPL mutation (n: 23)	3181 ± 1494	915 ± 493	t = 3.473P < 0.001

Of the 20 women included in this study, five (25%) were pregnant. There were no significant differences between the 5 pregnant and 15 non-pregnant women with regards to total cholesterol, TGs, HDL cholesterol, non-HDL cholesterol, LDL cholesterol, apo A1, apo B, lipoprotein (a), 25-OH vitamin D, and BMI. Only 1 pregnant patient had diabetes mellitus, and her HbA1c was 5.4%. These data are presented in Table 5. 

**Table 5:** Median and IQR biochemical data of the pregnant and nonpregnant patients.

Variables	Median [IQR] or mean ± SD data of patients with LPL mutation before TPE (n: 8)	Median [IQR] or mean ± SD data of patients without LPL mutation before TPE (n: 23)	Statistical test and P-values
Total cholesterol (mg/dL)	413 ± 227	475 ± 199	t = 0.736P = 0.468
TGs (mg/dL)	2990 ± 1496	3181 ± 1494	t = 0.311P = 0.758
HDL cholesterol (mg/dL)	29 [16–45]	30 [21–44]	U = 89.000P = 0.892
Non-HDL cholesterol (mg/dL)	356 [199–495]	404 [274–631]	U = 73.000P = 0.391
LDL cholesterol (mg/dL)	50 ± 28	99 ± 52	t = 2.567P = 0.017
Apo A1 (mg/dL)	104 [87–121]	112 [81–134]	U = 74.000P = 0.760
Apo B (mg/dL)	88 ± 34	113 ± 34	t = 1.761P = 0.090
Lipoprotein (a) (nmol/L)	9 [7–30]	14 [9–31]	U = 58.500P = 0.271
25-OH vitamin D (ng/mL)	14 [7–20]	15 [11–22]	U = 77.000P = 0.498
BMI (kg/m2)	24.8 ± 4.6	27.8 ± 5.9	t = 1.262P = 0.217

When the data of the male and female patients were compared, the only significant difference was between their apo A1 levels (P = 0.012). These data are presented in Table 6. 

**Table 6:** Median and IQR biochemical data of the female and male patients.

Variables	Median [IQR] data of the pregnant patients before TPE (n: 5)	Median [IQR] data of the non-pregnant patients before TPE (n: 15)	Statistical test and P-values
Total cholesterol (mg/dL)	325 [265–425]	415 [293–537]	U = 28.000P = 0.445
TGs (mg/dL)	2556 [1995–3430]	2828 [1571–3527]	U = 33.000P = 0.735
HDL cholesterol (mg/dL)	23 [19–41]	31 [21–40]	U = 34.000P = 0.760
Non-HDL cholesterol (mg/dL)	302 [246–385]	381 [270–506]	U = 27.000P = 0.359
LDL cholesterol (mg/dL)	69 [44–87]	65 [48–106]	U = 35.000P = 0.827
Apo A1 (mg/dL)	148 [111–244]	116 [99–133]	U = 14.500P = 0.151
Apo B (mg/dL)	108 [62–155]	99 [81–134]	U = 28.000P = 1.000
Lipoprotein (a) (nmol/L)	9 [7–37]	17 [9–37]	U = 18.000P = 0.287
25-OH vitamin D (ng/mL)	16 [11–21]	14 [10–20]	U = 32.500P = 0.662
BMI (kg/m2)	25.8 [22–31]	24.9 [19–28]	U = 29.000P = 0.458

Of the 31 patients included in this study, twenty (64.5%) were diabetic. The mean HbA1c value of the diabetic patients was 8.9 ± 2.6%. There was no significant difference in the average TG levels between the diabetic and non-diabetic patients (P = 0.905). None of the diabetic patients had ketoacidosis. However, there was a significant difference between the diabetic and nondiabetic patients in terms of BMI (P = 0.007). These data are shown in Table 7. 

**Table 7:** Median [IQR] or mean ± SD biochemical data of the diabetic and nondiabetic patients.

Variables	Median [IQR] or mean ± SD data of the female patients before TPE (n: 20)	Median [IQR] or mean ± SD data of the male patients before TPE (n: 11)	Statistical test and P-values
Total cholesterol (mg/dL)	416 ± 176	537 ± 237	t = 1.626P = 0.115
TGs (mg/dL)	2860 ± 1450	3626 ± 1446	t = 1.407P = 0.170
HDL cholesterol (mg/dL)	30 [22–40]	30 [13–56]	U = 101.000P = 0.710
Non-HDL cholesterol (mg/dL)	336 [271–495]	519 [282–691]	U = 73.000P = 0.127
LDL cholesterol (mg/dL)	75 ± 42	107 ± 62	t = 1.685P = 0.103
Apo A1 (mg/dL)	119 [103–141]	83 [77–110]	U = 37.500P = 0.012
Apo B (mg/dL)	107 ± 42	103 ± 19	t = –0.278P = 0.783
Lipoprotein (a) (nmol/L)	14 [8–14]	11 [7–18]	U = 72.500P = 0.399
25-OH vitamin D (ng/mL)	15 [10–20]	14 [11–23]	U = 103.000P = 0.772
BMI (kg/m2)	25.9 ± 6.4	28.9 ± 3.9	t = 1.410P = 0.169

The mean hospital stay of the patients was 14 ± 6 days (range: 4–29 days). There were no new local pancreatic complications or mortality observed during the follow-up. 

## 4. Discussion 

Insulin and heparin can be used to increase LPL activity during the acute phase in patients with pancreatitis secondary to HTG [4]. In order to reinforce treatment, antilipidemic medication must be administered using fibrates as a first choice. Nicotinic acid and omega-3 fatty acid can also be included in the treatment [4]. With patients that do not respond to treatment with current therapies, apheresis is among the options to reduce TG levels in the blood, allowing the application of TPE. TG levels can be reduced by 50%–80% with TPE [5]. In the current study, the TG decrease due to TPE was 69.7%. In the subgroups, TGs were reduced by 71.3% in those with diabetics, 64.5% in women, 77.2% in men, 67.3% in pregnant women, and 71.2% in those with LPL mutations. It was hypothesized that there was less of a reduction in TGs in women because estrogen is known to accelerate TG synthesis. 

The decline in TGs due to TPE was acute but transient, and should be supported by other medical treatments, even though current data has indicated that TG levels were unresponsive to other treatments. The ultimate goal in these patients was to use TPE to lower the TG level below 500 mg/dL. To protect against hypoglycemia during insulin infusion, nondiabetic individuals were recommended to undergo simultaneous dextrose infusion and frequent blood glucose measurements. TPE can be performed every 2–4 weeks, if oral medication is inadequate, following the regression of the clinical symptoms of pancreatitis [7]. The use of apheresis in the treatment of HTG was first reported by Betteridge et al. in 1978 [10]. Aside from case reports on the use of apheresis for the treatment of HTGP, the largest previously published series was with 20 cases by Chen et al. [11]. The current study of 31 cases was the largest series with HTGP. 

According to the American Association for Apheresis, therapeutic apheresis for HTGP is categorized as category III [7]. Apheresis category III includes disorders for which the optimum role of apheresis therapy has not been established. Decision making should be individualized. Thus, although there have been no recommendations for plasmapheresis in these patients, this information was based on expert opinions from case reports. There are many available therapies for significant HTG, including diet, oral lipid-lowering agents, IV insulin, heparin, and TPE [12]. The final choice of therapy for HTGP may be influenced by the cost, side effects, and availability of current therapies. Therefore, a controlled trial evaluating the efficacy of intravenous insulin, heparin, and TPE may helpful for determining the best choice for the treatment of acute pancreatitis secondary to HTG. Frankova et al. reported no superiority of either IV insulin or apheresis for the treatment of severe HTG among patients hospitalized for pancreatitis [13]. In addition, the study of Soyaltın et al. could not determine any benefit to apheresis treatment over other treatments in terms of hospital stay, mobility, and mortality [14]. It has been reported that acute pancreatitis has a mortality of up to 17% if it necrotizes [15]. TPE was initially used as the first step in the treatment of HTGP in order to prevent the pancreatitis from necrotizing, as well as to prevent organ failure. Apheresis may confer an additional benefit by removing proteases associated with acute pancreatitis [16]. 

Since the treatment modality may vary based on the type of mutation, the mutation responsible for primary HTG should be investigated after the exclusion of secondary causes. For example, fish oil supplements are effective in the treatment of excessive hepatic lipase production disorders, but are ineffective and contraindicated in LPL deficiency, due to their potential to contribute to an increase in chylomicron. Further, drugs that reduce lipid levels, such as fibrates, are useful for treating other lipid metabolism disorders, but are generally ineffective in cases with LPL deficiency [17]. The most frequently reported causes of primary HTG are LPL mutation, apo C-II deficiency, lecithin cholesterol acyl transferase deficiency, apoC-III overexpression, apoAV deficiency, excessive synthesis of angiopoietin-like protein 3, cholesterol ester storage disease, and antibody deficiency against glycosylphosphatidylinositol-anchored high-density lipoprotein-binding protein 1 [1,4]. In the current study, 8 of the 31 HTG patients (25.8%) had an LPL mutation; however, other genetic mutations were not investigated. This work had some limitations because it was performed retrospectively; hence, the clinical severity of pancreatitis could not be reliably reported.

In conclusion, TPE is a safe, fast, and effective method used in experienced centers [11,16,18]. Based on the literature and results obtained from the current study, it was concluded that TPE is an effective alternative treatment option for patients with HTGP, as it leads to a fast and reliable reduction in TG levels.
